# Application of PVDF Organic Particles Coating on Polyethylene Separator for Lithium Ion Batteries

**DOI:** 10.3390/ma12193125

**Published:** 2019-09-25

**Authors:** Yuan Wang, Chuanqiang Yin, Zhenglin Song, Qiulin Wang, Yu Lan, Jinpeng Luo, Liwen Bo, Zhihao Yue, Fugen Sun, Xiaomin Li

**Affiliations:** Institute of Photovoltaics, Nanchang University, Nanchang 330031, China; paul1042400516@163.com (Y.W.); cqyin@ncu.edu.cn (C.Y.); 15095815440@163.com (Z.S.); wql1751791764@163.com (Q.W.); lanyuncu@163.com (Y.L.); 15053980225@163.com (J.L.); 13767406553@163.com (L.B.); yuezhihao@ncu.edu.cn (Z.Y.); sunfugen@163.com (F.S.)

**Keywords:** PVDF, polyolefin separator, lithium ion batteries, organic coating, electrolyte wettability, electrochemical performance

## Abstract

Surface coating modification on a polyethylene separator serves as a promising way to meet the high requirements of thermal dimensional stability and excellent electrolyte wettability for lithium ion batteries (LIBs). In this paper, we report a new type of surface modified separator by coating polyvinylidene fluoride (PVDF) organic particles on traditional microporous polyethylene (PE) separators. The PE separator coated by PVDF particles (PE-PVDF separator) has higher porosity (61.4%), better electrolyte wettability (the contact angle to water was 3.28° ± 0.21°) and superior ionic conductivity (1.53 mS/cm) compared with the bare PE separator (51.2%, 111.3° ± 0.12°, 0.55 mS/cm). On one hand, the PVDF organic polymer has excellent organic electrolyte compatibility. On the other hand, the PVDF particles contain sub-micro spheres, of which the separator can possess a large specific surface area to absorb additional electrolyte. As a result, LIBs assembled using the PE-PVDF separator showed better electrochemical performances. For example, the button cell using a PE-PVDF as the separator had a higher capacity retention rate (70.01% capacity retention after 200 cycles at 0.5 C) than the bare PE separator (62.5% capacity retention after 200 cycles at 0.5 C). Moreover, the rate capability of LIBs was greatly improved as well—especially at larger current densities such as 2 C and 5 C.

## 1. Introduction

Lithium ion batteries (LIBs), which possess several advantages such as high specific energy, low self-discharge, good cycling performance, no memory effect and green environmental protection, are becoming very promising efficient secondary batteries and the fastest developing chemical energy storage power supply [1,2]. At present, LIBs have successfully replaced traditional lead acid batteries as energy storage devices for digital equipment such as mobile phones and laptops [3]. However, there are still many problems to overcome, such as long-term cycle stability, capacity retention rate, charging and discharging at high C rates and safety concerns when they are applied to hybrid electric vehicles and aerospace equipment [4]. 

As one of the key components, a separator plays a crucial role in the performance of LIBs and is called the “third pole” of LIBs as well. On the one hand, the separator acts as the barrier that separates the positive and negative electrodes; this ensures the safety of LIBs during application. On the other hand, it is a porous membrane that allows for the rapid transportation of lithium ions between the positive and negative electrodes during electrochemical charge-discharge processes [5,6]. Current commercial LIB separators mainly include wet and dry laying polyolefin separators, such as polyethylene (PE) and polypropylene (PP). Polyolefin separators can be widely used in LIBs due to their good mechanical strength, good thermal shutdown properties, excellent electrochemical stability and low price. However, commercial polyolefin separators suffer from thermal contraction at higher temperatures; this causes some safety problems for LIBs. Meanwhile, polyolefin separators have poor wettability to the electrolytes and this severely limits the long-term life cycle of LIBs and its capacity retention at high rates [7,8].

In view of the current shortcomings of commercial polyolefin separators used in LIBs, efforts including coating separators, organic/inorganic composite separators, new material system separators, etc., have been proposed [9]. Inorganic ceramic coating polyolefin separators such as silicon dioxide (SiO_2_) [10,11], aluminium oxide (Al_2_O_3_) [12,13] and boehmite (AlOOH) [14] are the most effective methods for surface modification of polyolefin separators. Inorganic nano-ceramic particles featuring high melting points and hardness can significantly improve thermal dimensional stability and mechanical strength of the separator after coating. However, the weak interfacial bonding between the inorganic coating layer and the organic polyolefin causes the inorganic coating layer to easily fall off during use of the battery, thereby blocking the micropores of the polyolefin separator [12]. 

Polyvinylidene fluoride (PVDF), a semi-crystalline polymer, has low hardness, electrochemical stability and good affinity to electrolytes. PVDF polymer chains contain strong electron-withdrawing groups (–C–F–) and have a high dielectric constant (ε = 8.4), which is beneficial to promote more complete dissolution of lithium salt and increase carrier concentration [15]. As an important polymer material for LIBs, PVDF is often used as the binder for anode and cathode preparations. Furthermore, it is becoming a good organic coating choice for traditional polyolefin separators as well due to its special functional group structure [16]. Li et al. [17] reported the preparation of a porous PVDF separator with a thickness of ~31 µm using a traditional phase inversion process. In comparison with the polypropylene separator Celgard 2500, the porous PVDF separator displayed a higher porosity (56%), a better liquid electrolyte uptake (200%) and electrochemical stability under 5 V. Kim et al. [18] researched a porous PVDF separator prepared by electrospinning. Their results showed that PVDF separators had high porosity and ionic conductivity; when the average fiber diameter was 0.45 to 1.38 μm, the apparent porosity and ionic conductivity were 80% to 89% and 1 × 10^−3^ S/cm, respectively. However, there are some disadvantages such as high crystallinity limiting the transport of lithium ions through the separator and weak mechanical strength of the electrospinning fiber to hinder further development of PVDF as a matrix separator in LIBs. Alcoutlabi et al. [19] used an electrospinning PVDF nanofiber on the surface of the Celgard 2400 polypropylene microporous separator, which can balance the advantages of a traditional polyolefin separator and the PVDF polymer. In addition, the surface of the electrospun composite separators of PVDF nanofibers had better adhesion with the electrode. To the best of our knowledge, it is difficult to industrialize the separator prepared by electrospinning technology, and oil-soluble solvents such as dimethylacetamide (DMAc) are not environmentally friendly. 

In this paper, PVDF organic particles were utilized to modify polyolefin PE separators by a simple spreader coating process. In addition, water was used as the solvent, and BYK (Germany Beck Chemical Co., Bad Homburg, Germany) additives served as the water-based assistant during the preparation of the separator. PVDF particles not only improve organic compatibility between the organic PVDF polymer and the electrolyte, but possess a larger specific surface area to retain additional electrolyte as well. It can significantly improve electrolyte retention of the separator and the lithium ionic conductivity, which helps to improve electrochemical performances‑especially the capacity retention and C rate capacity.

## 2. Experimental

### 2.1. Preparation of PE-PVDF Separator

The coating slurry was supported by dispersing polyvinylidene fluoride particles (PVDF, LBG, Arkema, Paris, France) using a mixed solution of deionized water and an auxiliary agent such as an ammonium acrylate type dispersing agent (BYK LPC 22136, ALTANA, Bad Homburg, Germany) or a silicone surfactant type wetting agent (BYK-LPX20990, ALTANA, Bad Homburg, Germany). Bentonite clay (Laponite RD, ALTANA, Bad Homburg, Germany) served as the anti-settling agent and the polyacrylate binder (BYK-LPC22346, ALTANA, Bad Homburg, Germany) was ball milled for 2 h at 400 r/min. The slurry was applied to both sides of the 20 µm thick PE separator (SK Innovation, Seoul, South Korea) by dip coating. After that, air and moisture inside the micropores of the separator were completely removed by drying for 6 h in a blast air and vacuum oven at 60 °C. The bare PE separator and the PE coated PVDF organic particle separator were marked as bare PE separator and PE-PVDF separator, respectively.

### 2.2. Cell Preparation and Assembly

The coin-type half cells of CR2032 being tested were used to investigate the influence of different separators for batteries. For the half-cell preparation, the cathode was composed of 80 wt.% active material LiNi_0.3_Co_0.3_Mn_0.3_O (NCM-111, theoretical capacity of 278 mAh/g), 10 wt.% conductive agent super-P and 10 wt.% of a PVDF binder used as a working electrode with Li metal serving as a counter electrode. The above working electrode with a load of 42.7 g/m^2^ was cut into a wafer (14 mm diameter) and assembled in a glove box into a battery (NCM-111|separator soaking electrolyte|Li metal) together with a Li metal, different separators and a liquid electrolyte (1 M LiPF_6_ in EC:DEC:DMC = 1:1:1 *v*/*v*).

### 2.3. Characterization of the PE-PVDF Separator

The microscopic surface and cross-sectional morphologies of the untreated PE and the PE-PVDF separators were investigated by Field Emission Scanning Electron Microscopy (FE-SEM, JSM 6701F, JEOL Techniques, Tokyo, Japan). The thickness of the sample was measured by a digital display thickness gauge. Mechanical strength, including tensile and puncture strength, were measured on the intelligent electronic tension machine (XLM, Labthink, Jinan, China) at a stretching rate of 25 mm/min, for which the separators were rectangular shaped (8 cm × 2 cm). The thermal stability of the separators (19 mm diameter) was investigated by observing their dimensional changes as compared to original samples after heating at 130, 140, 150 and 160 °C for 30 min; shrinkage was calculated using the following Equation (1):

(1) $$Shrinkage\;(\%)=\frac{S_{0}-S_{T}}{S_{0}}\times 100\%$$

where *S*_0_ and *S_T_* correspond to the separator areas before and after thermolysis, respectively.

Electrolyte wettabilities of the separator samples was examined by contact angle measurements by dropping water on the surface of the sample and taking an optical photograph after 30 s using an optical contact-angle measuring instrument (DSA100, Kruss, Hamburg, Germany). The porosity of the bare PE separator and the PE-PVDF separator were investigated by measuring the weight change of an original separator and one after full saturation in n-hexadecane for 6 h, and calculated with Equation (2):

(2) $$Porosity\;(\%)=\frac{m_{1}-m_{0}}{\rho_{n}v}\times 100\%$$

where *m*_0_ and *m*_1_ indicate the weights of the separators before and after saturation in n-hexadecane, respectively. *ρ_n_* and *v* refer to the density of n-hexadecane and the total volumes of n-hexadecane and the PE separator. Electrolyte uptakes of the bare PE separator and the PE-PVDF separator were determined using Equation (3):

(3) $$Electrolyte\;uptake=\frac{m_{1}-m_{0}}{m_{0}}\times 100\%,$$

where *m*_0_ and *m*_1_ refers to the weights of the separators before and after soaking in a liquid electrolyte for 6 h.

### 2.4. Electrochemical Performance Evaluation

Ionic conductivities of the samples were measured by electrochemical Impedance Spectroscopy (EIS). The cells assembled by sandwiching the separator soaking in liquid electrolyte between two stainless steel (stainless steel|separators soaking electrolyte|stainless steel) of different separators was scanned in frequency range of 1 × 10^6^ Hz to 1 Hz with amplitude of 10 mV. Then, calculation of ionic conductivity ran as follows:

(4) $$\sigma =\frac{d}{R_{b}\times s},$$

where *R_b_* refers to the bulk resistance and *d* and *S* correspond to the separator’s thickness and size, respectively 

Electrochemical stabilities were characterized by performing a linear sweep voltammetry at 5 mV/s from 2.5 to 6 V using batteries assembled by sandwiching the separator soaking in liquid electrolyte between the stainless and Li metal (stainless steel|separators soaking electrolyte|Li metal) of the various separators. The EIS spectra of cells (NCM-111|separators soaking electrolyte|Li metal) for different separators were gained in frequency range of 100 KHz to 0.1 Hz with an amplitude of 5 mV after 0.1 C pre-treatment.

The coin-type cell (NCM-111|separators soaking electrolyte|Li metal) was used to investigate the influence of the PVDF coating layer for electrochemical properties. After assembling the half cells, they were set aside for 12 h to allow the separators to fully integrate with the electrolyte. To evaluate the stability during a charge-discharge test cycle at high current density, the cells containing different separators were cycled 200 times in a potential window from 2.8 to 4.3 V at 0.5 C in the Neware battery test system. The rate capabilities of the half cells were evaluated by subsequently five cycles at a variety of discharging current densities (0.2, 0.5, 1, 2, 3, 5 and 0.2 C).

## 3. Results and Discussion

**Figure 1:**
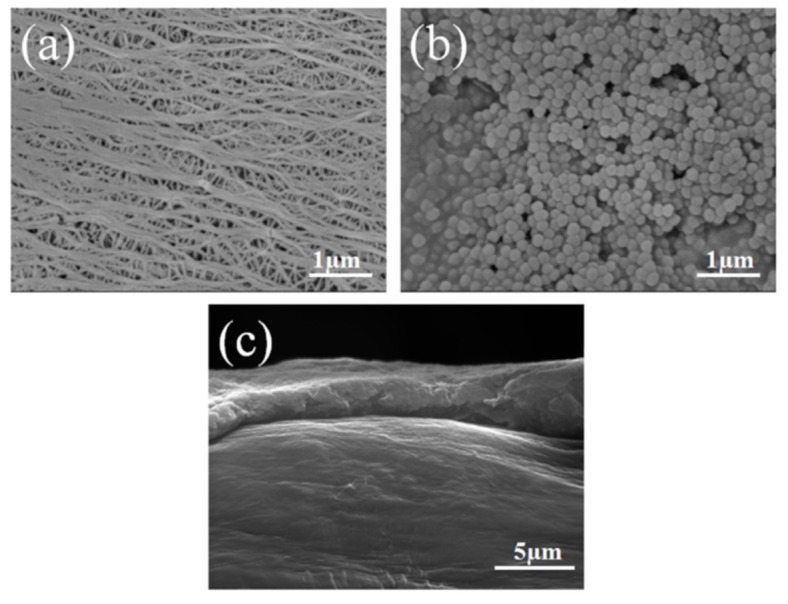
SEM photographs of (**a**) the bare PE separator; (**b**) the PE-PVDF separator; (**c**) the cross section of PE-PVDF separator.

SEM was used to evaluate the morphologies of the bare PE and PE-PVDF separators, as shown in Figure 1. A typical PE separator during the wet process is shown in Figure 1a and exhibits a large number of interconnected submicron pore structures to serve as storage spaces to absorb a significant amount of electrolyte and to provide channels for lithium ions passing through during the LIBs charge/discharge process. The surface morphology of the PVDF with the organic particle coating is presented in Figure 1b. A mass of PVDF spherical particles with diameters of ~100 nm are uniformly distributed on the surface of the PE separator matrix, forming many microporous structures, which greatly increases the electrolyte storage capacity for the PE-PVDF separator. 

For separators used in LIBs, an appropriate coating thickness can provide more space for electrolyte storage while minimizing the adverse impact on the internal resistance of the battery often observed upon increasing the coating thickness [20]. Good interfacial adhesion between the coating layer and the matrix separator can effectively improve the mechanical properties of the composite separator and prevent the coating layer from falling off during charge and discharge of LIBs [21,22]. Figure 1c illustrates a cross-sectional image of the PE-PVDF separator. It was found that the tight combination was present at the interface between the coating layer and the PE matrix with a thickness of approximately 2 to 3 µm. Suitable thicknesses for LIBs separators and excellent adhesive properties between the coating layer and the PE matrix are required for increasing the comprehensive performance. It is obvious that the morphology of the PE-PVDF separator is beneficial to the application in LIBs.

**Figure 2:**
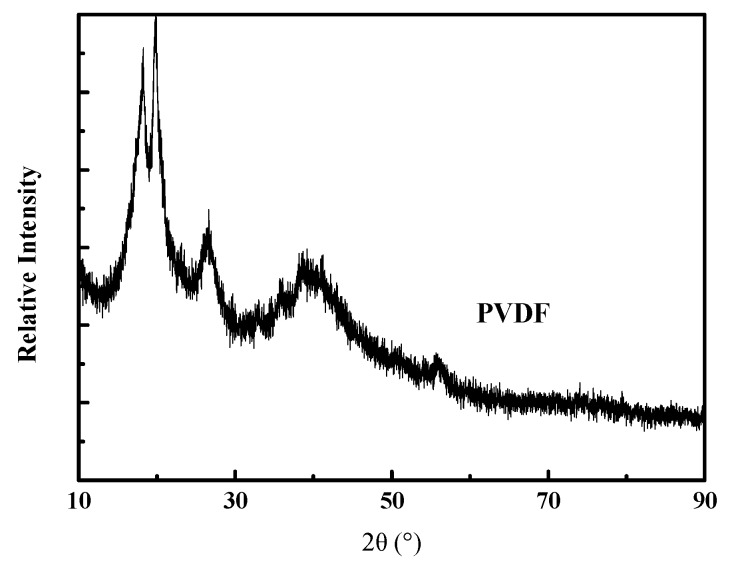
XRD curve of PVDF particles.

To understand the mechanism of the PVDF coating layer influence on separator performance, the evaluation of PVDF organic particles on physical and chemical properties were carried out. As shown in Figure 2, the X-ray diffraction (XRD) curve of PVDF organic showed the degree of crystallinity. To the best of our knowledge, the peaks at 18.25, 20.08, and 26.78° refer to α-type, β-type and ƴ-type crystals, which are the most common forms of PVDF crystals [17]. The test data of XRD curve was fitted with Jada software to obtain the crystallinity of PVDF organic particles, as shown in Table 1. It is evident that PVDF organic particles used in the experiment for a typical semi-crystalline polymer (crystallinity degree, 55.39%). Other physical and chemical properties, such as the melting point (151–157 °C), strength at yield (35 MPa) and elongation at break (>400%) are shown in Table 1 as well. In summary, the PVDF used in the experiment is a semi-crystalline polymer with low mechanical strength but high toughness.

**Figure 3:**
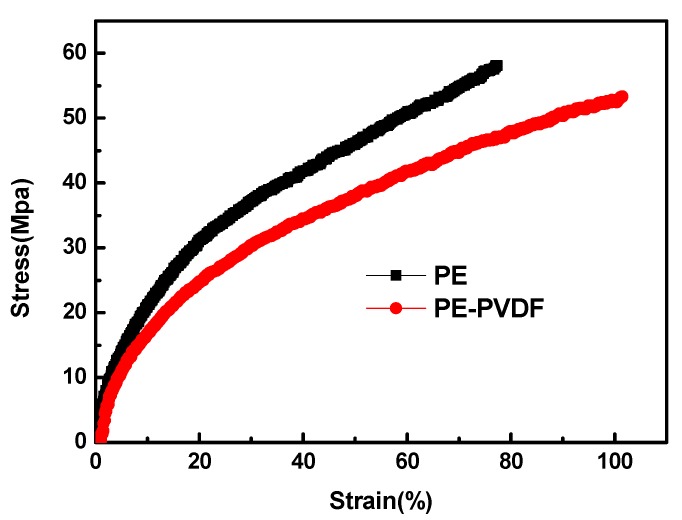
The stress-strain curves of bare PE and PE-PVDF separators.

The thickness of the PE-PVDF separator was measured using a digital thickness gauge and is shown in Table 2. Compared with the bare PE separator, the coating layer on the PE-PVDF separator has a uniform thickness of 2–3 µm, which is coincident in the cross-section image result shown in Figure 1c. Mechanical performances including tensile strength, elongation at break and puncture strength are important parameters associated with the safety performance of LIBs. The separator should possess sufficient strength to accommodate the mechanical stresses generated by LIBs, in particular, cases such as lithium dendrites growing to pierce the separator and reducing chemical and side reactions of the positive and negative electrodes of the LIBs. In the charge/discharge processes of LIBs, the separator is readily damaged by lithium dendrites, which are formed from the lithium metal cathode material, and leads to internal short circuits [4]. As shown in Table 2, compared with the bare PE separator with the tensile strength of 58.1 MPa, the PE-PVDF separator has a smaller tensile strength of 55.35 MPa, while the tensile elongation at break is higher. Table 2 clearly shows that the tensile elongation at break of PE-PVDF separator is 101.4%, 1.5 times greater than that of the bare PE separator (77.4%). It is no doubt that the above results of mechanical properties are mainly attributed to the organic polymer coating layer on the PVDF. The mechanical strength of the organic polymer is mainly affected by the crystalline degree, therefore, the semi-crystalline nature of the PVDF polymer suggests that the mechanical strength of the composite separator cannot be significantly improved [23]. Although the tight combination was observed at the interface of the PVDF coating layer and the PE matrix separator as shown in Figure 1c, the adverse impact of increasing the thickness of the PE-PVDF separator overshadowed the incremental increase of the composite separator’s mechanical strength. Therefore, the tensile strength of the PE-PVDF separator decreases relative to the mechanical strength of the bare PE separator. A molecular chain with strongly flexible functional groups (–C–F–) in the PVDF amorphous region can greatly improve elongation at the break of the PE-PVDF separator, which partially explains the larger fracture energy of the composite separator, as shown in Figure 3 and Table 1 [17]. Furthermore, the penetration strength of the bare PE and PE-PVDF separators are of 5.67 and 6.20 N, respectively. 

**Figure 4:**
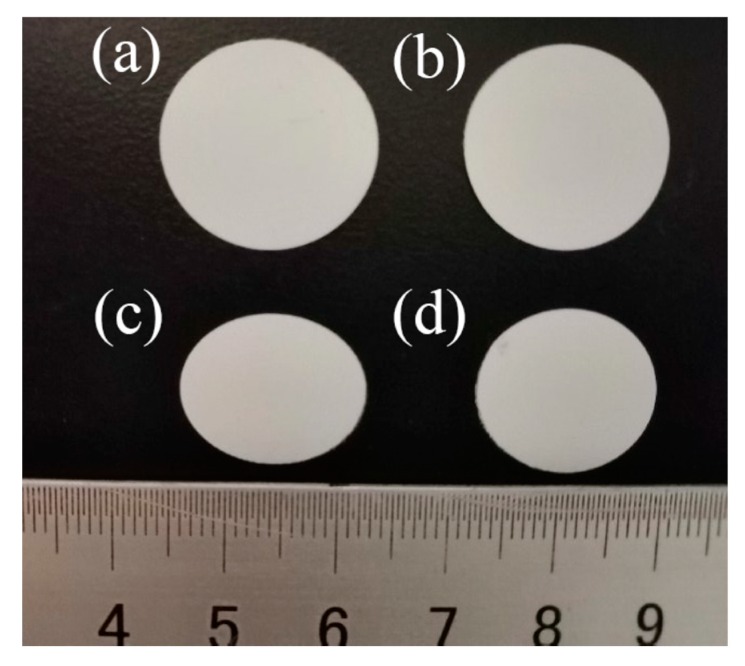
Digital camera images of (**a**,**b**) bare PE and PE-PVDF separator at room temperature (25 °C); (**c**,**d**) bare PE separator and PE-PVDF separators (140 °C/30 min).

To further evaluate the influence of PVDF organic particles on the thermal stability of the separator, bare PE and PE-PVDF separators were treated at 130, 140, 150 and 160 °C for 30 min, respectively. Figure 4a,b shows the original dimensions of the bare PE and the PE-PVDF separators prior to thermolysis. Figure 4c,d shows the pictures of the bare PE and the PE-PVDF separators after heating at 140 °C for 30 min. The thermal shrinkage of the PE separator after heating at 140 °C was 52.3%; the PE-PVDF separator fared slightly better at 48%. The main reason that PVDF coating layer does not significantly improve the thermal stability of the PE-PVDF composite is that PVDF is a type of polymer as well with a lower melting point (171–175 °C, Table 1) than a ceramic coating separator [21]. As the heating temperature increased, the bare PE separator shows drastic shrinkage and appears transparent.

**Figure 5:**
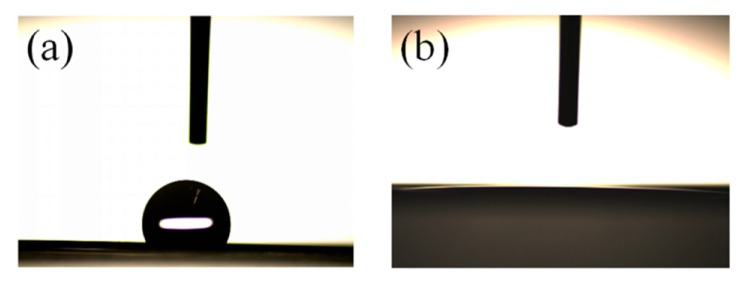
Contact angle photographs of (**a**) bare PE separator; (**b**) PE-PVDF separator.

A contact angle measurement was carried out to study the electrolyte wettability for PE and PE-PVDF separators and are shown in Figure 5a,b and Table 3. The PE-PVDF separator has a much lower contact angle of 3.28° ± 0.21° than the bare PE separator (111.3° ± 0.12°) and is attributed to the lyophilic performance of the PVDF polymer coating on the surface of PE, which possesses similar intermolecular properties as the organic carbonates in the liquid electrolyte, indicating better organic compatibility compared with the bare PE separators [16]. 

Porosity is interpreted as the percentage of the micropore volume in the separator to the total volume of the separator and is an important parameter affecting its electrochemical performance. As shown in Table 3, the porosity of the PE-PVDF separator (61.4%) is larger than that of the bare PE separator (51.2%); this is due to the large specific surface area of the PVDF coating layer, which can provide additional voids for the PE-PVDF separator, as shown in Figure 1b. The PE-PVDF separator has higher porosity and better compatibility with the liquid electrolyte and is beneficial for the electrolyte uptake rate. The electrolyte uptake of the PE-PVDF separator reached levels as high as 208%, higher than the bare PE separator (167%; Table 3). Electrolyte uptake has a significant influence on the lithium ion transportation between the anode and cathode and further impacts the electrochemical performance of the LIBs [24]. 

**Figure 6:**
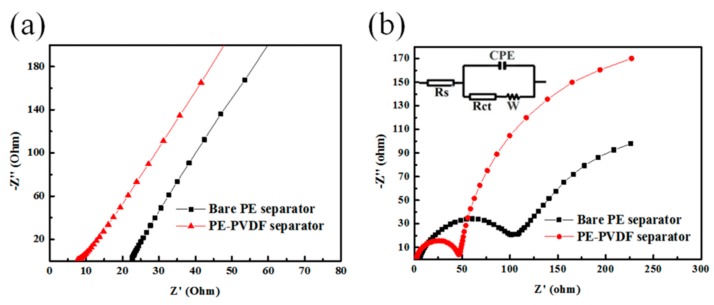
(**a**) Ionic conductivities of the liquid electrolyte-soaking separators; (**b**) Nyquist plots of cells containing bare PE and PE-PVDF separators after pre-cycling at 0.1 C.

Ionic conductivity is an important and basic property for LIB separators and is obtained from Nyquist plots of the cell (stainless steel|separators soaking electrolyte| stainless steel), as shown in Figure 6a. Bulk resistance (*R_b_*) and sample ionic conductivities are shown in Figure 6a with the values listed in Table 4. The intercepts on the real axis denotes the bulk resistance (*R_b_*) with values of 18.18 and 8.173 Ω for the bare PE and PE-PVDF separators, respectively. Equation (4) has been used to calculate the ionic conductivity and the conductivity of the PE-PVDF separator was 1.53 mS/cm, nearly three times greater than the bare PE separator (0.55 mS/cm). The better wettability to electrolyte and higher porosity produced by interconnected and porous surface morphology of the PVDF coating layer significantly improved the ionic conductivity of the PE-PVDF separator. 

The electrochemical performance of the cells before and after surface modification of PVDF needs additional study. Figure 6b shows the Nyquist plots of the cells (NCM-111|separators soaking electrolyte|Li metal) containing the bare PE and the PE-PVDF separators after pre-cycling at 0.1 C with AC impedance measurement. It shows as well that the equivalent circuit in the inset of Figure 6b is in accord with electrochemical impedance spectroscope (EIS) analysis results. Generally speaking, the AC impedance spectrum of the cells includes the bulk resistance indicated by the high frequency x-axis intercepts, two partially overlapped semicircles representing lithium ion migration through the solid electrolyte interface (SEI) named *R_SEI_* and the formation of ions at the interface between the electrode and the electrolyte named charge transfer resistance *R_ct_* in the middle and low frequency region, respectively. In addition, the straight slopping line at the low frequency end corresponds to the diffusion of ions in the electrode material [7,19]. As shown in Table 4, the *R_ct_* of the cell employing the PE-PVDF separator has a lower charge transfer resistance with the value of 33.49 Ω, compared with that of containing the bare PE separator whose *R_ct_* value was 75.33 Ω. The liquid electrolyte uptake rate of the LIB separators increased, and favoured the charge transport of lithium ions on the surface of the electrode and the electrolyte; both of which are beneficial to electrochemical performance improvement [25,26]. The cells employed the PE-PVDF separator have a smaller *R_ct_* due to its significant enhancement of the electrolyte uptake. 

**Figure 7:**
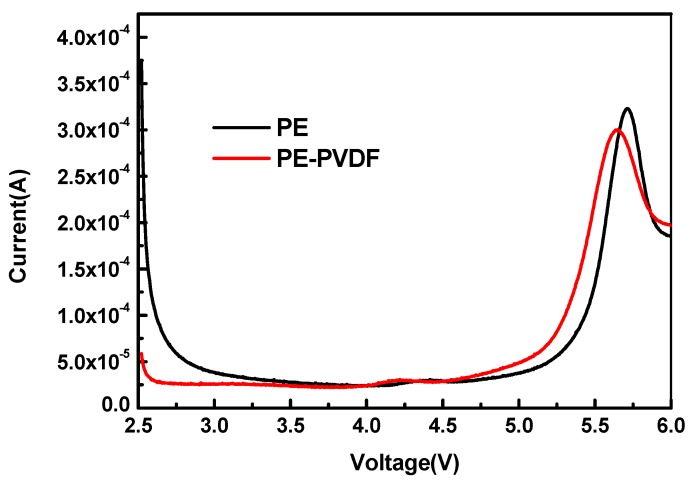
Linear sweep voltammetry of bare PE and PE-PVDF separators.

To evaluate the influence of the PVDF organic coating layer for electrochemical stability of the cells (stainless|separators soaking electrolyte|Li metal), linear sweep voltammetry curves (LSVs) were carried out at room temperature (25 °C). As shown in Figure 7, it features LSVs of the cells utilizing different separators with a voltage range from 2.5–6.0 V. There were no current changes during the potential sweeps under 5.0 V for both samples, which indicates that both cells are stable up to 5.0 V. When the sweeping voltage increased from 5.0 V to 6.0 V, there was an obvious oxidation peak that appeared in the LSVs for both the bare PE and the PE-PVDF separators, which indicated the current change was caused by electrolyte decomposition [27]. The similar LSVs of the bare PE and the PE-PVDF separators indicate that the PVDF organic coating layer has no negative impact on the electrochemical stability of LIBs. The oxidation peak of the PE-PVDF separator appears at a slightly lower potential but is smoother than the PE separator; this means the PVDF-PE has a wider working window [28] and the PE-PVDF separator is very suitable for application in LIBs [18]. 

**Figure 8:**
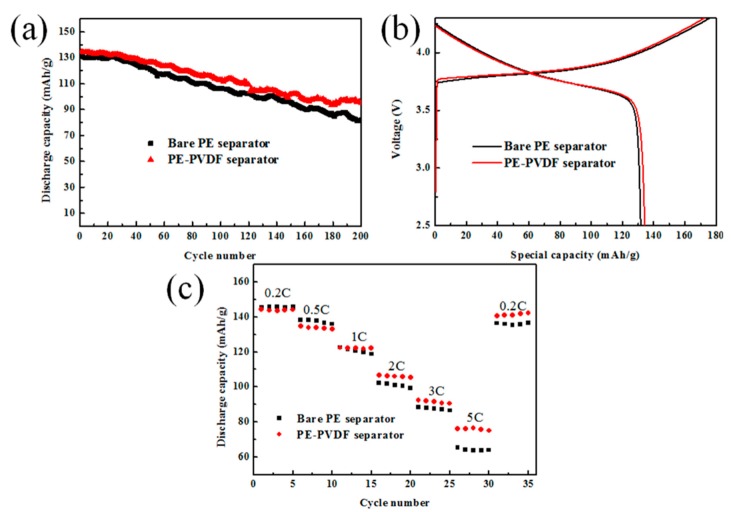
(**a**) The cycle performances; (**b**) first charge-discharge curve; (**c**) rate capabilities of the cells with bare PE and PE-PVDF separators.

To study the influence of the PVDF on the long-term cycling stabilities of LIBs, the CR2036 coin-type half cells (NCM-111|separator soaking electrolyte|Li metal) were charged and discharged 200 times in voltage range of 2.5 to 4.3 V and current density of 0.5 C. Cycling stabilities of the half cells assembled by PE and PE-PVDF separators are shown in Figure 8a. After 200 cycles, the discharge specific capacity of the cell containing bare PE separator is attenuated from 131.62 to 82.29 mAh/g, which means that the capacity retention rate of the cell was 62.5%, while the discharge specific capacity of the cell using the PE-PVDF separator decreased from 134.60 to 95.11 mAh/g with a capacity retention of 70.1%. Those results suggest the cell using the PE-PVDF separator had a better capacity retention ratio and a higher first charge-discharge specific capacity. In order to verify electrical performance repeatability, five cells assembled using the same sample were tested simultaneously. Those results showed that the cell assembled PVDF coating PE separator has excellent repeatability; the maximum deviation of capacity retention after 200 cycles was only ~5%. The first charge-discharge curves at 0.5 C are shown in Figure 8b. The cell containing the PE-PVDF separator has a higher first charge-discharge efficiency (77.8%), compared to the bare PE separator (74.9%). There are two main reasons for the above result: on one hand, the PVDF coating layer has good organic compatibility with the liquid electrolyte and has a larger electrolyte uptake rate of 208%. On the other hand, PVDF can contact the electrolyte and form a kind of gel electrolyte, which accelerates the migration of lithium ions in the pores of the separators to some extent [29,30]. 

Further evaluation of the PVDF coating layer was carried out in terms of the C-rate capabilities of the half cells (NCM-111|separators soaking electrolyte|Li metal) which used different separators. As described in Figure 8c, the cells containing the bare PE separator and the PE-PVDF separator were charged-discharged continuously at different current densities of 0.2, 0.5, 1, 2, 3 and 5 C, and then back to 0.2 C for five cycles at each C-rate. For both half cells containing the bare PE separator and the PE-PVDF separator, the discharge specific capacity of the cells gradually decreased as the current density increased due to incomplete lithium ion intercalation and deintercalation into the crystal lattice of the negative and positive electrode materials [31]. The first discharge capacities of cells containing the bare PE separator and the PE-PVDF separator were 145.69 and 144.27 mAh/g at 0.2 C, respectively. When the current densities were increased to 1, 2, 3 and 5 C, the first discharge specific capacities of the cells with the bare PE separators were 122.58, 102.22, 88.58 and 64.56 mAh/g; the capacity retention rates were 96.90%, 97.13%, 97.84% and 97.4%. However, the first discharge specific capacities of the cells with the PE-PVDF separators were 122.43, 106.50, 92.34 and 75.86 mAh/g; the capacity retention rates were 99.67%, 99.07%, 98.02% and 98.82%. Obviously, the discharge capacity of the cell using the bare PE separator was slightly higher than that of the PE-PVDF separator at a lower charge density (0.2 C). However, when the charge-discharge ratios of the cells were increased to 2 and 3 C, the cell containing the PE-PVDF separator had higher discharge specific capacities and charge capacity retention rates. At the current density of 5 C, this advantage was even more significant. The better retention of the electrolyte for the PVDF coating layer helps explain the above results, which significantly impacts ionic conductivities as shown in Figure 6—especially at high C rates [22]. In conclusion, the rate capabilities of the bare PE and PE-PVDF separators align well with the ion conductivity values and electrolyte uptakes as discussed above. Thus, the PE-PVDF separator makes for a good choice for LIB applications due to its excellent rate capacity property and optimized electrochemical stability.

## 4. Conclusions 

In this paper, a new type of water-based PVDF organic particle coating PE separator was successfully prepared for high-power Li-ion battery (LIB) applications. Molecular structures that contain strongly electron withdrawing functional groups (–C–F–) for the PVDF coating layer are favourable to electrochemical performance of LIBs. The PVDF organic particle has good organic compatibility and significantly improves the wettability of the PE-PVDF separator to the liquid electrolyte. Compared with the bare PE separator (111.3° ± 0.12°), the PE-PVDF separator has a smaller wetting angle with water (3.28° ± 0.21°). Furthermore, the spherical PVDF particles with a larger specific surface area greatly increase the contact area between the PE-PVDF separator and the electrolyte. Due to its larger ionic conductivity and liquid uptake rate with the value of 1.53 mS/cm and 204%, respectively, LIBs using PE-PVDF separators possess better first charge efficiency and capacity retention rates. Moreover, LIBs using the PE-PVDF separator have better discharge capacities at large current densities (2, 3 and 5 C). The commercial polyolefin separator modified by PVDF organic particles is a promising way to improve the electrochemical performance of LIBs—especially in the field of high-power applications.

## Figures and Tables

**Table 1:** Physical and chemical properties of PVDF.

Crystallinity	Melting Point (°C)	Strength at Yield (MPa)	Elongation at Break (%)
Semi-crystalline	151–157	35	>400

**Table 2:** Thickness, tensile strength, puncture test and elongation data of separator samples.

Parameters	Bare PE Separator	PE-PVDF Separator
Thickness (µm)	20	25–26
Tensile strength (MPa)	58.1	55.35
Puncture test (N)	5.67	6.20
Tensile elongation at break (%)	77.4	101.4

**Table 3:** Porosity, electrolyte uptake and contact of separator samples.

Parameters	Bare PE Separator	PE-PVDF Separator
Contact angle	111.3° ± 0.12°	3.28° ± 0.21°
Porosity (%)	51.2	61.4
Electrolyte uptake (%)	167	208

**Table 4:** Ionic conductivities and impedances of bare PE and PE-PVDF separators.

Sample	*R_b_* (Ω)	Ion Conductivity (mS cm^−1^)	*R_ct_* (Ω)
Bare PE separator	18.19	0.55	75.33
PE-PVDF separator	8.173	1.53	33.49
